# Non-invasive mechanical ventilation as an adjunct to pulmonary rehabilitation in patients with bronchiectasis: a pathophysiological perspective

**DOI:** 10.3389/fresc.2026.1887626

**Published:** 2026-07-15

**Authors:** Jhojan Sebastian Herrera-Vargas, Cristian Jose Gomez-Vega

**Affiliations:** 1 Faculty of Respiratory Therapy, Institución Universitaria Visión de las Américas, Medellín, Colombia; 2 Adult Intensive Care Unit, Clínica Somer, Rionegro, Colombia

**Keywords:** bronchiectasis, exercise tolerance, noninvasive ventilation, rehabilitation, respiratory mechanics

## Abstract

Bronchiectasis is a complex respiratory disease characterized by irreversible bronchial dilatation, mucus hypersecretion, and impaired mucociliary clearance. These structural changes result in a predominantly obstructive pattern that increases airway resistance, impairs gas exchange, and leads to air trapping and dynamic hyperinflation. This scenario increases the work of breathing (WOB) and places the inspiratory muscles at a mechanical disadvantage, precipitating muscle fatigue during exercise and limiting the potential benefits of pulmonary rehabilitation (PR). In this context, non-invasive mechanical ventilation (NIV) emerges as an intervention with high biological plausibility as an adjunct during physical training. Its mechanism of action is based on the application of inspiratory positive pressure to overcome resistive load and expiratory pressure to counteract hyperinflation, thereby optimizing ventilatory efficiency. Although the efficacy of NIV in improving exercise tolerance is well documented in chronic obstructive pulmonary disease (COPD), in bronchiectasis the evidence is still incipient and largely based on extrapolation; therefore, it is imperative to conduct research to define its efficacy and safety in order to improve functional prognosis in this population.

## Introduction

Bronchiectasis is currently recognized as a complex condition requiring a multidisciplinary approach. It is defined by permanent and irreversible bronchial dilatation, frequently associated with chronic cough and sputum hypersecretion that perpetuates the cycle of infection and inflammation (1–3). These structural changes not only compromise airway integrity but also alter ventilatory mechanics, increasing resistive load and the work of breathing, thereby contributing to a progressive decline in the patient's functional capacity (4).

In advanced stages, these alterations impair alveolar ventilation and lead to hypercapnic respiratory failure, in a pattern similar to that observed in COPD (5). In this context, conventional management is based on airway clearance strategies and PR as first-line interventions (5–8). However, when ventilatory load exceeds the patient's compensatory capacity, NIV becomes central. Although recent guidelines from the European Respiratory Society support its use in patients with rapid symptom deterioration or exacerbations, there is growing interest in its application outside the acute setting, specifically as a support to maximize rehabilitation outcomes (6, 9).

This transition from the use of NIV as a rescue therapy toward its use as an exercise-facilitating tool represents a paradigm shift in the management of bronchiectasis. Despite this potential, knowledge gaps remain regarding its efficacy and safety as an adjunct to PR. In particular, evidence on selection criteria, titration parameters during exercise, and therapeutic targets is still limited. In this context, the present article proposes a perspective focused on the role of NIV as an adjunct to pulmonary rehabilitation, analyzing from a pathophysiological standpoint how this technology may mitigate dyspnea and respiratory failure and improve physical performance, integrating the available evidence with the challenges for its safe implementation.

## Alterations in ventilatory mechanics in bronchiectasis

From a structural standpoint, bronchiectasis is characterized by abnormal and irreversible dilatation of the airways; however, functionally, it predominantly behaves as an obstructive disorder. Recent data indicate that the obstructive pattern is the most prevalent (51.6%), and it is directly associated with greater severity in Bronchiectasis Severity Index and FACED scores, as well as higher levels of dyspnea (10).

This obstruction, which paradoxically occurs in dilated bronchi, can be explained by *Poiseuille's law*. Transmural inflammation and mucin hypersecretion (MUC5B and MUC5AC), which alters the rheological properties of mucus, reduce the effective luminal radius through edema and mucus plugging (5, 11–13). Because airway resistance is inversely proportional to the fourth power of the radius, small reductions due to thick mucus result in disproportionate increases in resistive load, particularly in the small airways (11). Airway obstruction is not uniform; this heterogeneity, which can be objectively assessed by volumetric capnography through an increased phase III slope, leads to uneven ventilation distribution. Consequently, a ventilation–perfusion (V/Q) mismatch occurs, which explains the reduced diffusing capacity for carbon monoxide (DLCO) observed in approximately 56% of patients (12, 14). Furthermore, even in individuals with normal spirometry, the presence of “slow emptying units” contributes to reduced ventilatory efficiency.

**Figure 1:**
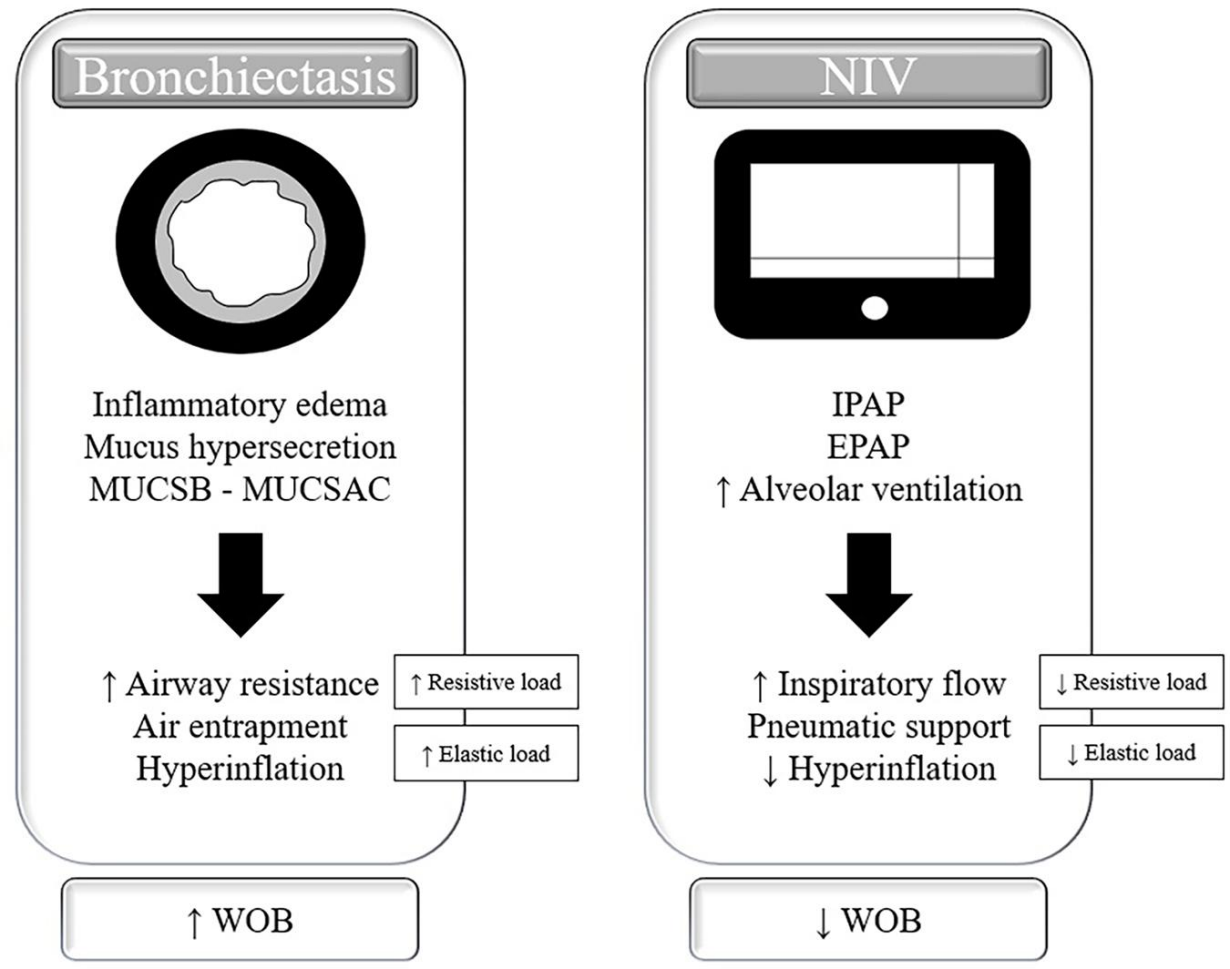
Physiopathological impact of bronchiectasis on WOB and its modulation by NIV. Bronchiectasis is characterized by mucus hypersecretion and airway inflammation, leading to increased airway resistance, air trapping, and dynamic hyperinflation, which collectively raise the WOB through increased resistive and elastic loads. NIV, through IPAP and EPAP, reduces inspiratory effort, improves alveolar ventilation, and provides pneumatic splinting of the airways, thereby decreasing both resistive and elastic loads and ultimately reducing WOB. NIV, non-invasive ventilation; IPAP, inspiratory positive airway pressure; EPAP, expiratory positive airway pressure; WOB, work of breathing. Prepared by the authors.

The most critical consequence of this obstruction is premature closure of alveolar units. Plethysmographic data confirm that the most frequent abnormality is not obstruction *per se*, but air trapping, present in 70% of patients (residual volume >120% of predicted) (10, 12, 14). This phenomenon results in dynamic hyperinflation, which during exercise or rehabilitation shifts lung volumes toward less compliant regions of the pressure–volume curve (15), as illustrated in Figure 1.

From a physiological perspective, this increases the elastic component of the WOB. The respiratory muscles, already weakened compared to healthy individuals, are required to generate higher pressure gradients under a mechanical disadvantage (muscle fibers shortened by hyperinflation), which precipitates muscle fatigue and ventilatory failure (4, 15–17).

Considering the above, patients enrolled in PR programs present a baseline mechanical disadvantage characterized by dynamic hyperinflation and air trapping, which is further exacerbated during rehabilitation-related interventions, both in functional assessments (such as the 6-minute walk test [6MWT], recognized as a clinically relevant indicator of functional capacity) and in therapeutic airway clearance strategies (18). Although these interventions are essential to optimize mucociliary clearance and improve physical function, they entail an additional increase in WOB, already elevated at baseline due to airflow obstruction and inspiratory muscle impairment. Therefore, PR must be strictly individualized, as the increased resistive and elastic load, together with the shift of lung volume toward less compliant regions, may promote the onset of exercise-induced muscle fatigue.

## Physiological rationale of NIV in the management of WOB in bronchiectasis

To mitigate the increase in respiratory work, patients should be incorporated into respiratory therapy and PR programs, focused on optimizing mucociliary clearance and secretion removal, with the aim of improving ventilation and gas exchange efficiency (7–9). NIV, in turn, plays a limited but specific role in bronchiectasis, mainly in patients with acute and/or chronic respiratory failure in advanced stages (5–8, 17).

From a biophysical standpoint, NIV operates through a transairway positive pressure gradient that modifies intrathoracic fluid mechanics (19, 20). The programmed inspiratory positive airway pressure (IPAP) assumes a fraction of the resistive load, generating the flow required to overcome the impedance of obstructed airways without requiring extreme pleural pressure deflection by the patient. Simultaneously, expiratory positive airway pressure (EPAP) acts as a pneumatic support that counteracts air trapping, preventing dynamic collapse of the small airways and mitigating hyperinflation (19, 21). This support shifts the inspiratory operating point toward a region of greater compliance on the pressure–volume curve, reducing the elastic component of the work of breathing. Consequently, by unloading the diaphragm and accessory respiratory muscles, NIV preserves the length–tension relationship of muscle fibers (avoiding critical shortening due to hyperinflation), thereby optimizing ventilatory efficiency, reducing the oxygen cost of breathing, and expanding the functional reserve required for rehabilitation (19, 20) (Figure 1).

## Role of NIV in bronchiectasis

The application of NIV in the context of bronchiectasis has been primarily delineated in three clinical scenarios. First, it is considered as support in the management of acute respiratory failure secondary to severe exacerbations (7, 16, 22). Second, it is used in the management of chronic hypercapnic respiratory failure in the home setting, with the aim of improving gas exchange (23–25). Finally, a less explored but emerging scenario is the use of NIV as support during physical training in PR programs, aiming to mitigate muscle fatigue (9).

Unlike what has been observed in COPD, where NIV during exercise has been extensively studied, the evidence in bronchiectasis is scarce and is mainly based on physiological rationale and extrapolations from other obstructive diseases (17, 26). Theoretically, the use of ventilatory support during exertion aims to mitigate the load on respiratory muscles and reduce sensory dyspnea, which could act as a “bridge” allowing patients with severe impairment to tolerate higher training intensities (9, 17). However, current guidelines emphasize that, although this approach is physiologically plausible for improving exercise tolerance in hypercapnic patients, there are no large-scale randomized clinical trials validating its additional benefit over conventional PR (8). Therefore, its implementation remains conditioned by the limited robustness of the available data, which are predominantly derived from small observational studies (27).

When extrapolating evidence from similar scenarios, it is observed that NIV during physical training in patients with COPD, particularly in severe to very severe stages (GOLD III–IV), allows the achievement of higher exercise intensities and, consequently, greater physiological adaptations compared with unsupported training (27, 28). A recent meta-analysis (35 studies, *n* = 1,321) demonstrated that NIV is associated with significant improvements in 6-minute walk test (6MWT) distance, maximal work rate, endurance time, peak oxygen uptake (VO₂ peak), and ventilatory efficiency, along with a reduction in lactate levels (27). Consistently, a systematic review published by the Cochrane group reported increases in peak exercise capacity (17%) and endurance (59%), explained by unloading of the respiratory muscles, optimization of gas exchange, and attenuation of dynamic hyperinflation (with increases in inspiratory capacity of 0.19 to 0.22 L) (28). Although these findings cannot be directly extrapolated to bronchiectasis, they provide indirect support for the physiological rationale of NIV in this setting. In particular, attenuation of dynamic hyperinflation may be relevant, as air trapping and dynamic hyperinflation are common pathophysiological features in bronchiectasis (10, 12, 14). Nevertheless, the realization of these potential benefits depends on adequate technical implementation of ventilatory support. In particular, appropriate interface selection, correct titration of ventilatory parameters, and optimization of humidification and thermal conditioning of the inspired gas are key elements to ensure tolerance, effectiveness of support, and patient adherence during exercise (29–31).

Despite these findings in COPD, the effects on quality of life are less consistent, and the overall quality of evidence remains moderate. In the specific case of bronchiectasis, the absence of equivalent studies leaves a critical knowledge gap. This scientific gap represents an opportunity for pulmonologists, respiratory therapists, and rehabilitation specialists to investigate whether the benefits of NIV observed in other obstructive diseases are reproducible in patients with bronchiectasis within the context of pulmonary rehabilitation, whose lung mechanics and hypersecretion profile present unique challenges.

## Potential risks

As with any ventilatory support intervention the application of NIV in ventilatory failure or during exercise requires careful consideration of its physiological limitations and associated risks. In patients with bronchiectasis the substantial mucus burden may not only compromise interface seal integrity and patient tolerance but also predispose positive-pressure airflow to promote retrograde secretion displacement or mucus plugging secondary to luminal impaction (32). These risks may be further exacerbated when inadequate gas conditioning is provided, as insufficient humidification and heating of inspired air can increase mucus viscosity, impair mucociliary transport, and promote airway secretion retention. Consequently, airway resistance may increase, the WOB may rise, and the effectiveness of diaphragmatic unloading may be reduced, thereby limiting ventilatory performance and optimal gas exchange during physical exertion.

Furthermore, the therapeutic effectiveness of NIV depends on adequate patient–ventilator interaction and optimal synchronization between respiratory demand and ventilator assistance. Patient–ventilator dyssynchrony, together with unintentional air leaks and increased instrumental dead space, may substantially impair effective alveolar ventilation (33). Although the incidence of hemodynamic and parenchymal adverse events remains low when ventilator settings are carefully individualized, excessive IPAP or inappropriate EPAP levels may result in alveolar overdistension, increasing the risk of volutrauma, barotrauma, and impaired venous return (19, 20). Therefore, the successful integration of NIV within a PR program requires careful patient phenotyping, individualized titration of ventilatory pressures, and continuous monitoring of respiratory mechanics and cardiopulmonary interactions.

## Discussion

The integration of NIV as an adjunct in respiratory rehabilitation for patients with bronchiectasis represents a therapeutic frontier with biological plausibility, but still lacking definitive clinical validation. From a pathophysiological perspective, NIV has the capacity to counteract the high resistive load imposed by edema and secretions, as well as to compensate for the elastic component derived from dynamic hyperinflation. The main contribution of NIV lies in its ability to simultaneously modulate the resistive and elastic components of WOB, which are significantly increased in this population (4, 12, 14).

IPAP reduces the resistive load by generating a pressure gradient that facilitates inspiratory flow through airways partially obstructed by secretions and inflammatory edema. This mechanism decreases the need to generate high negative pleural pressures, thus reducing the effort of the inspiratory muscles (19, 20, 34). In parallel, EPAP helps counteract the dynamic collapse of the small airways, a key phenomenon in the pathophysiology of air trapping and dynamic hyperinflation (21, 35). These effects promote a redistribution of lung volume towards areas of greater compliance, reducing the elastic component of WOB (15, 19). This dual effect could be particularly relevant during PR and exercise in patients with bronchiectasis, where dynamic hyperinflation tends to worsen, displacing the diaphragm into shortened positions that compromise its contractile efficiency.

NIV by providing pneumatic support, may help preserve the length–tension relationship of respiratory muscle fibers, potentially improving ventilatory efficiency and reducing the metabolic cost of breathing (19, 20). These effects could theoretically enhance oxygen delivery to peripheral muscles during exercise; however, whether this translates into clinically meaningful improvements in rehabilitation outcomes for patients with bronchiectasis remains unknown and requires confirmation in dedicated clinical trials.

Specific clinical evidence in bronchiectasis is scarce. Most of the knowledge comes from studies in COPD, where NIV during exercise has consistently demonstrated improvements in functional capacity, exercise tolerance, and physiological variables such as peak VO2 and ventilatory efficiency (27, 28). However, extrapolating these results should be done with caution, given that bronchiectasis presents distinctive pathophysiological characteristics, such as mucus hypersecretion, ventilatory heterogeneity, and a high prevalence of chronic infections (1, 12).

The lack of randomized clinical trials in bronchiectasis represents a critical limitation. Generating robust evidence should focus not only on functional outcomes, such as exercise capacity, but also on patient-centered variables, including dyspnea, quality of life, and exacerbation rate. Furthermore, it will be essential to evaluate the safety of NIV use during exercise, particularly in relation to the risk of barotrauma, ventilator-patient mismatch, and secretion retention.

## Data Availability

The original contributions presented in the study are included in the article/Supplementary Material, further inquiries can be directed to the corresponding author.
